# Antibacterial and antibiofilm potential of *Lacticaseibacillus rhamnosus* YT and its cell-surface extract

**DOI:** 10.1186/s12866-022-02751-3

**Published:** 2023-01-12

**Authors:** Chengran Guan, Wenjuan Zhang, Jianbo Su, Feng Li, Dawei Chen, Xia Chen, Yujun Huang, Ruixia Gu, Chenchen Zhang

**Affiliations:** Key Lab of Dairy Biotechnology and Safety Control, College of Food Science and Engineering, Yangzhou University, Yangzhou, Jiangsu China

**Keywords:** Antibacterial, Antibiofilm, *Lactobacillus rhamnosus* YT, Cell-surface extract, Physicochemical properties

## Abstract

**Background:**

Foodborne pathogens and spoilage bacteria survived in the biofilm pose a serious threat to food safety and human health. It is urgent to find safe and effective methods to control the planktonic bacteria as well as the biofilm formation. Substances with antibacterial and antibiofilm activity found in lactic acid bacteria were mainly metabolites secreted in the cell-free supernatant. Previously, *Lacticaseibacillus rhamnosus* YT was isolated because its cell pellets displayed distinguished antibacterial activity under neutral conditions. This study aimed to investigate the antibacterial and antibiofilm properties of the *L. rhamnosus* YT cells and its crude cell-surface extract.

**Results:**

The antibacterial activity of the *L. rhamnosus* YT cells constantly increased with cells growth and reached the peak value after the cells grew into stationary phase. After cocultivation with the *L. rhamnosus* YT cells, the biofilm formation of *B. subtilis* and *S. enterica* was reduced. The antibacterial activity of the *L. rhamnosus* YT cells was varied along with various culture conditions (carbon sources, nitrogen sources, medium pH and cultural temperatures) and the antibacterial intensity (antibacterial activity per cell) was disproportional to the biomass. Furthermore, the cell-surface extract was isolated and displayed broad antimicrobial spectrum with a bacteriostatic mode of action. The antibiofilm activity of the extract was concentration-dependent. In addition, the extract was stable to physicochemical treatments (heat, pH and protease). The extract performed favorable emulsifying property which could reduce the water surface tension from 72.708 mN/m to 51.011 mN/m and the critical micelle concentration (CMC) value was 6.88 mg/mL. Besides, the extract was also able to emulsify hydrocarbon substrates with the emulsification, index (E24) ranged from 38.55% (for n-hexane) to 53.78% (for xylene). The E24 for xylene/extract emulsion was merely decreased by 5.77% after standing for 120 h. The main components of the extract were polysaccharide (684.63 μg/mL) and protein (120.79 μg/mL).

**Conclusion:**

The properties of the extract indicated that it might be a kind of biosurfactant. These data suggested that *L. rhamnosus* YT and the cell-surface extract could be used as an alternative antimicrobial and antibiofilm agent against foodborne pathogens and spoilage bacteria in food industry.

**Supplementary Information:**

The online version contains supplementary material available at 10.1186/s12866-022-02751-3.

## Background

Foodborne disease is one of the most important public health issues around the world due to the ingestion of food contaminated by foodborne pathogens and spoilage bacteria [1]. Most of the foodborne bacteria survived in the shape of biofilm making planktonic bacteria aggregate, adhere to each other and encapsulate the bacteria in a structural colony [2]. Compared with the planktonic bacteria, biofilm make the bacteria 1000-fold more resistant to antibiotics and the immune system of the host which is critical and a matter of concern for many industries such as medical instrumentation, food, dairy, brewery, drinks and juices, aquaculture, *etc* [3, 4]. Therefore, to control food contamination caused by pathogens and spoilage bacteria, it is urgent to find safe and effective methods to control the planktonic bacteria as well as the biofilm formation.

Some lactic acid bacteria (LAB) with probiotic function and the GRAS (generally regarded as safe) status, are wildly used in food and pharmaceuticals industry [5]. These LAB has been shown to be effective strains for inhibiting foodborne pathogenic bacteria in numerous studies [6]. The substances with antibacterial activity were mainly organic acids, carbon dioxide, hydrogen peroxide, diacetyl, ethanol and bacteriocin [7, 8]. Nisin, produced by *Lactococcus lactis* ssp. *Lactis*, is highly active against Gram positive bacteria such as *Listeria monocytogenes*, *Staphylococcus aureus*, *Bacillus cereus*, *Lactiplantibacillus plantarum*, *Micrococcus luteus* and *Micrococcus flavus*. As the oldest known and most widely studied natural antibacterial bacteriocin, nisin is permitted as a safe food additive in over 50 countries around the world [9, 10].

Recently, LAB with both antibacterial and antibiofilm activity were found [11]. The cell-free supernatant of *Limosilactobacillus fermentum* TCUESC01 and *L. plantarum* TCUESC02 was demonstrated to inhibit the growth and the biofilm formation of *S. aureus* [12]. Exopolysaccharides produced by *L. plantarum* YW32 showed the ability to suppress the formation of biofilm by Gram-positive and negative pathogens [13]. To date, most of the substances with antibacterial and antibiofilm capabilities discovered in LAB were mostly metabolites secreted in the cell-free supernatant [14]. However, a few studies showed that substances on the surface of *Lactobacillus* also performed antibacterial and antibiofilm inhibition activities [15, 16]. For example, surface proteins containing cytoplasmic hydrolases from *L. acidophilus* inhibited *E. coli* growth by damaging the cell wall [17]. Jung et al. [18] showed lipophosphatidic acid of *L. plantarum* inhibited the biofilm formation of *Enterococcus faecalis* in a dose-dependent manner. *L. rhamnosus* cell-surface-derived biosurfactant displayed potent antiadhesion and antibiofilm ability by inhibiting the bacterial attachment to surfaces [19].

Studies have shown that the physicochemical properties of antibacterial agents varied widely among strains. Previously, *L. rhamnosus* YT was isolated in our lab because its cell pellets displayed distinguished antibacterial activity to Gram positive and negative spoilage bacteria. In this study, the antibacterial and antibiofilm properties of *L. rhamnosus* YT cells and the crude materials extracted from the cell surface were evaluated.

## Results

### Growth kinetics and antibacterial potential of *L. rhamnosus* YT cells

**Fig. 1:**
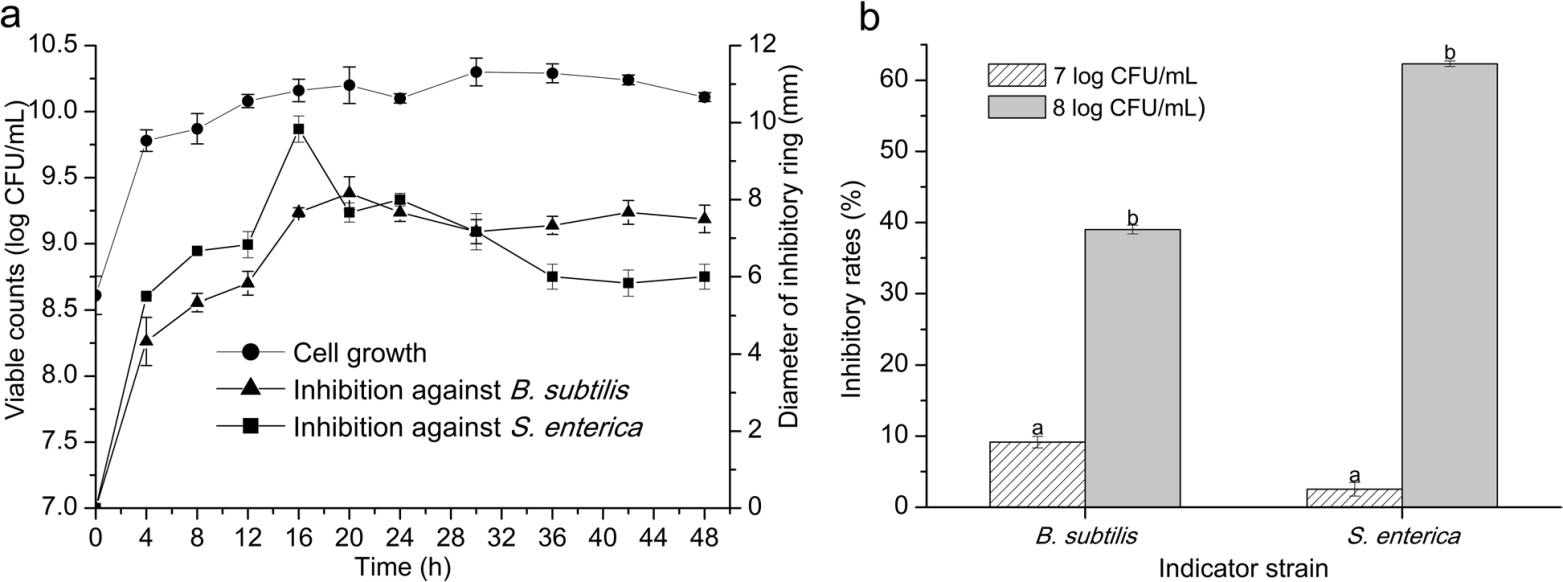
Growth profile of *L. rhamnosus* YT and its antibacterial and antibiofilm activity against *B. subtilis* and *S. enterica*. *L. rhamnosus* YT was cultivated and sampled at time intervals. The cell growth was measured and the antibacterial capacity of the cell pellets were assessed by the diameter of the inhibition zone on the plate (**a**). Inhibition of biofilm was proceeded with *L. rhamnosus* YT suspending in PBS buffer (10 mM, pH 7.0) (**b**)

*L. rhamnosus* YT was cultivated in the deMan, Rogosa and Sharpe (MRS) broth and sampled with specific time intervals. The cell pellets were obtained and resuspended in the phosphate buffered saline (PBS) buffer to measure the viable counts and the antibacterial/antibiofilm activity. The cell growth reached stationary phase with the highest biomass of 10.31 log CFU/mL after 20 h cultivation. Antibacterial activity of the cells was constantly increasing and the biggest diameter of inhibitory zone to *B. subtilis* and *S. enterica* was 8.17 mm at 20 h and 9.83 mm at 16 h, respectively (Fig. 1 a). Moreover, the biofilm formation of the indicator strains cocultured with *L. rhamnosus* YT was reduced (Fig. 1 b). The reduction rate was rising with the increase of cells concentration. With 8.0 log CFU/mL of *L. rhamnosus* YT, the formed biofilm of *B. subtilis* and *S. enterica* was reduced by 63 and 35%, respectively.

### Effect of culture conditions on the antibacterial activity of *L. rhamnosus* YT cells

To explore the factors affecting the antibacterial activity of the *L. rhamnosus* YT cells, the culture conditions including varied carbon sources, nitrogen sources, successive medium pH and temperatures were tested. After cultivation for 24 h, the cell pellets were separated for detection of viable counts and antibacterial activity.

**Fig. 2:**
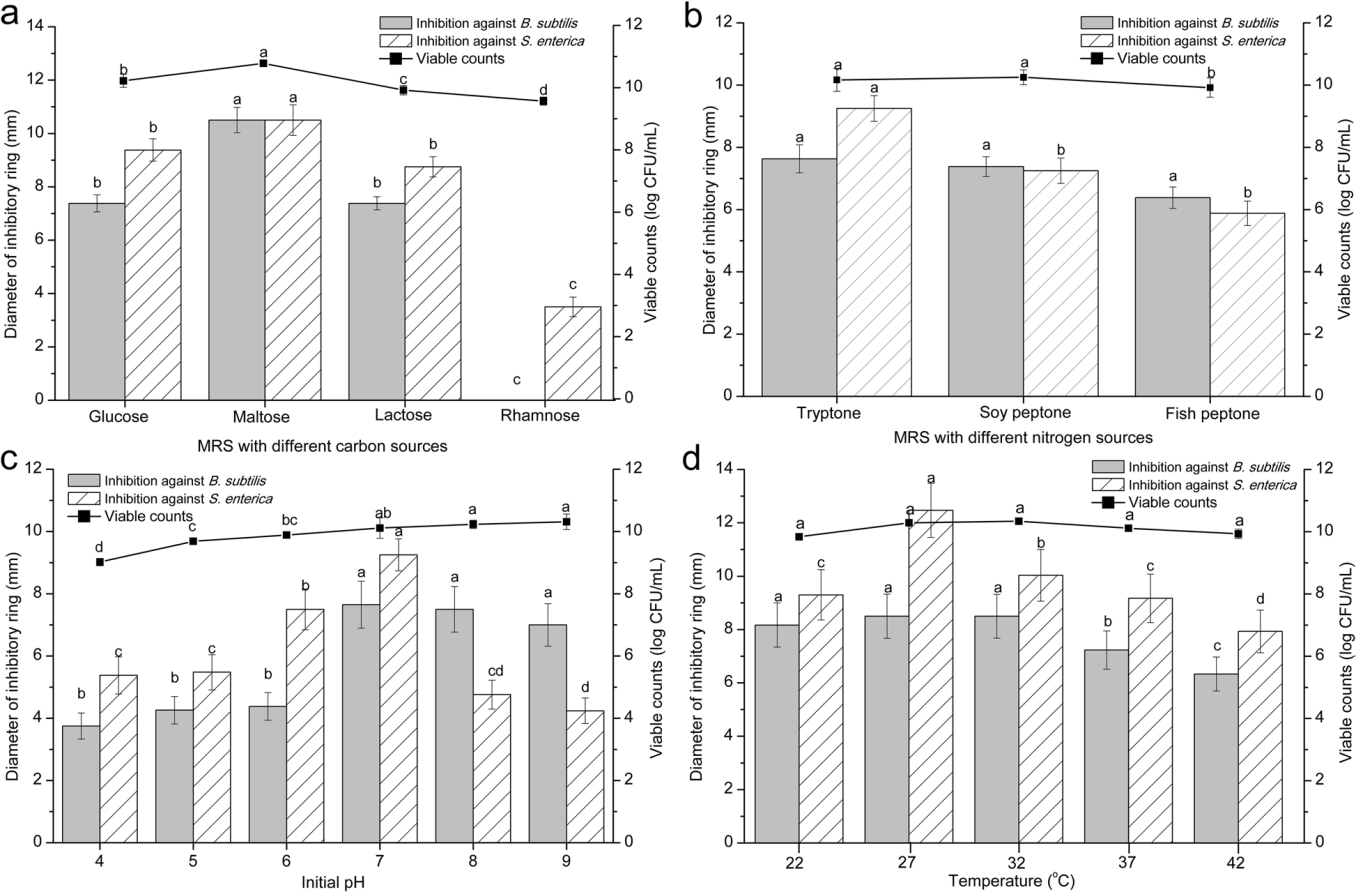
Effect of culture conditions on the biomass and antibacterial activity of *L. rhamnosus* YT. The culture conditions including varied carbon (**a**) and nitrogen (**b**) sources were tested at successive medium pH value (**c**) and temperatures (**d**). After cultivation for 24 h, the cell pellets were separated to detect the viable counts and antibacterial activity

Cultivating in the broth of MRS-glucose, MRS-maltose, MRS-lactose and MRS-rhamnose, the viable counts were varied from 9.57 log CFU/mL (with rhamnose) to 10.78 log CFU/mL (with maltose). The diameter of inhibitory zone to *B. subtilis* and *S. enterica* ranged from 0 mm to 10.5 mm and 3.5 mm to 10.5 mm, respectively. *L. rhamnosus* YT cultivated with glucose and maltose possessed similar biomass while the corresponding antibacterial activity was quite different. Furthermore, using rhamnose as the carbon source, *L. rhamnosus* YT cells performed very tiny inhibitory zone although the cell concentration was higher than 9.0 log CFU/mL (Fig. 2 a).

Cultivating in the MRS broth independently using tryptone, soy peptone and fish peptone as nitrogen sources, the viable counts were varied from 9.92 log CFU/mL (with fish peptone) to 10.25 log CFU/mL (with soy peptone). There was slight difference in the cell biomass and the antibacterial activity of *L. rhamnosus* YT cells cultivated with different nitrogen sources (Fig. 2 b).

To determine the effect of initial medium pH, *L. rhamnosus* YT was cultivated in the MRS broth with a series of initial pH values from 3.0 to 9.0. *L. rhamnosus* YT grew better when the initial medium pH value was higher or equal to 7.0. Meanwhile, the antibacterial activity of *L. rhamnosus* YT cells to *B. subtilis* and *S. enterica* was steadily enhanced with the pH value increasing from 4.0 to 7.0. Then as the pH value continually raised to 9.0, the antibacterial activity of *L. rhamnosus* YT basically kept stable against *B. subtilis* while significantly decreased against *S. enterica* (Fig. 2 c).

*L. rhamnosus* YT was cultivated at 22 °C, 27 °C, 32 °C, 37 °C and 42 °C, respectively. With temperature increasing, the biomass and the antibacterial activity showed similar variation of increasing first and then decreasing. The viable counts ranged from 9.84 log CFU/mL (at 22 °C) to 10.34 log CFU/mL (at 32 °C). The biggest inhibitory zone against *B. subtilis* and *S. enterica* was *L. rhamnosus* YT cultivated at 27 °C (Fig. 2 d).

### Extraction of the cell-surface antibacterial substances from *L. rhamnosus* YT and its antibacterial potential

**Fig. 3:**
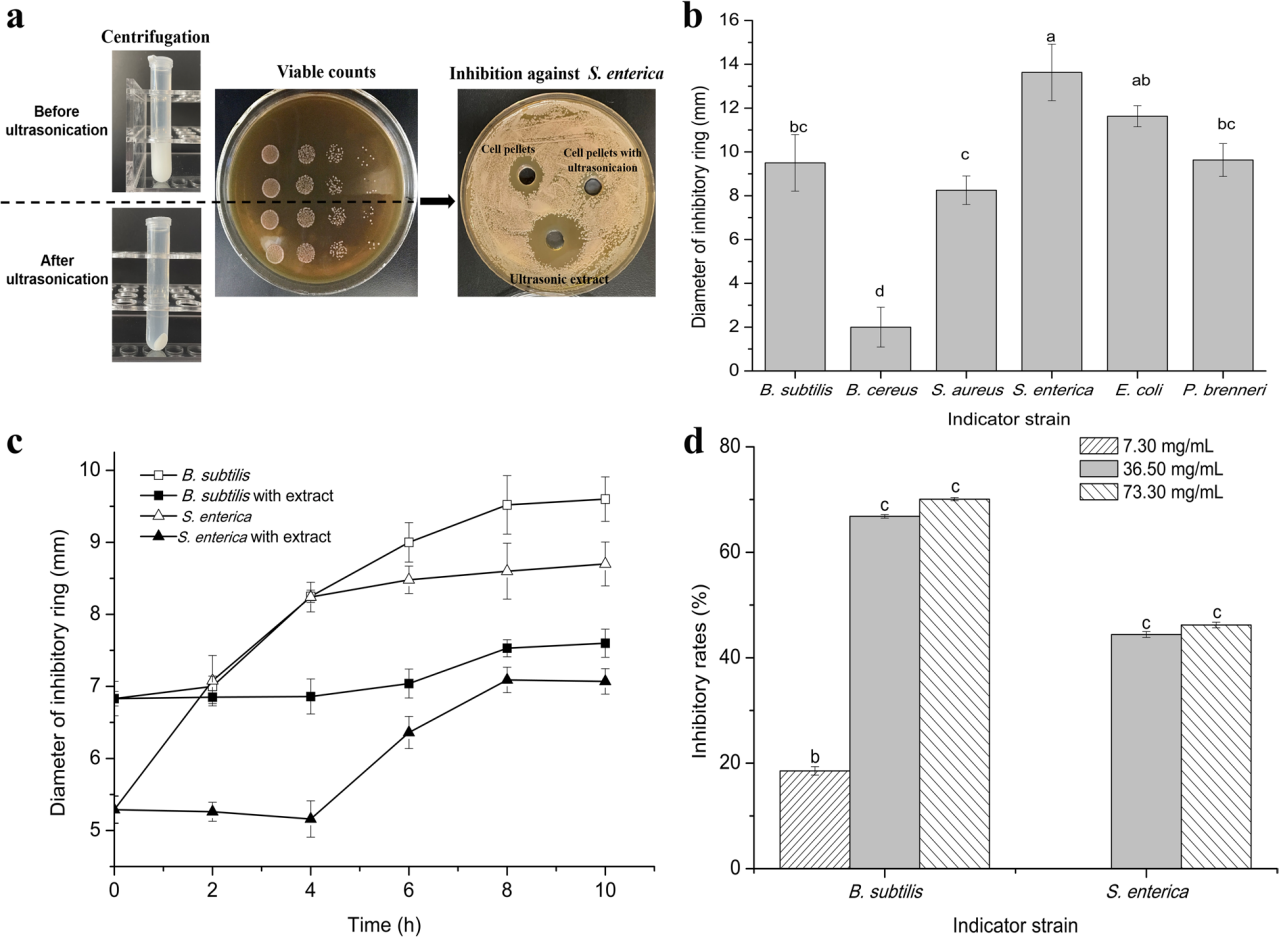
Extraction of the cell-surface antibacterial substances from *L. rhamnosus* YT (**a**) and its antibacterial spectrum (**b**), mode of action (**c**) and antibiofilm potential (**d**). Ultrasonic procedure was optimized to obtain the maximal amount of extract under the premise of keeping the cell integrity. After sonification and centrifugation, the viable counts and antibacterial activity were measured (**a**). Antibacterial ability of the extract to multiple bacteria was measured by agar well diffusion method (**b**). The extract concentrated at 15 mg/mL was cocultivated with indicator strains to analyze its antibacterial action mode (**c**). The antibiofilm potential of the extract with different concentration was determined (**d**)

Ultrasonication was employed to isolate the antibacterial substances from the cell surface of *L. rhamnosus* YT. Firstly, the ultrasonic procedure was optimized to avoid the leakage of the intracellular materials. After ultrasonication, the cell pellets were much easier to be compacted to be separated and the viable counts kept consistent suggesting the cell structure remaining intact. The ultrasonic extract displayed apparent bacteriostatic ring on the plate while the ultrasonicated cells without visible inhibitory zone (Fig. 3a). These results indicated that the antibacterial extract was merely obtained from the cell surface of *L. rhamnosus* YT.

The extract showed varied inhibitory capacity to typical spoilage bacteria detected in food contamination (Fig. 3 b). By co-culture with the extract, the lag growth phase of *B. subtilis* and *S. enterica* was obviously delayed by 2 h and 4 h, respectively. The viable counts of *B. subtilis* and *S. enterica* were independently decreased by 65.7 and 31.67% after cultivation for 10 h (Fig. 3 c). The biofilm formation of *B. subtilis* and *S. enterica* was significantly reduced with the ultrasound extract at 36.50 mg/mL and 73.30 mg/mL (Fig. 3 d).

### Properties of the cell-surface extract

**Fig. 4:**
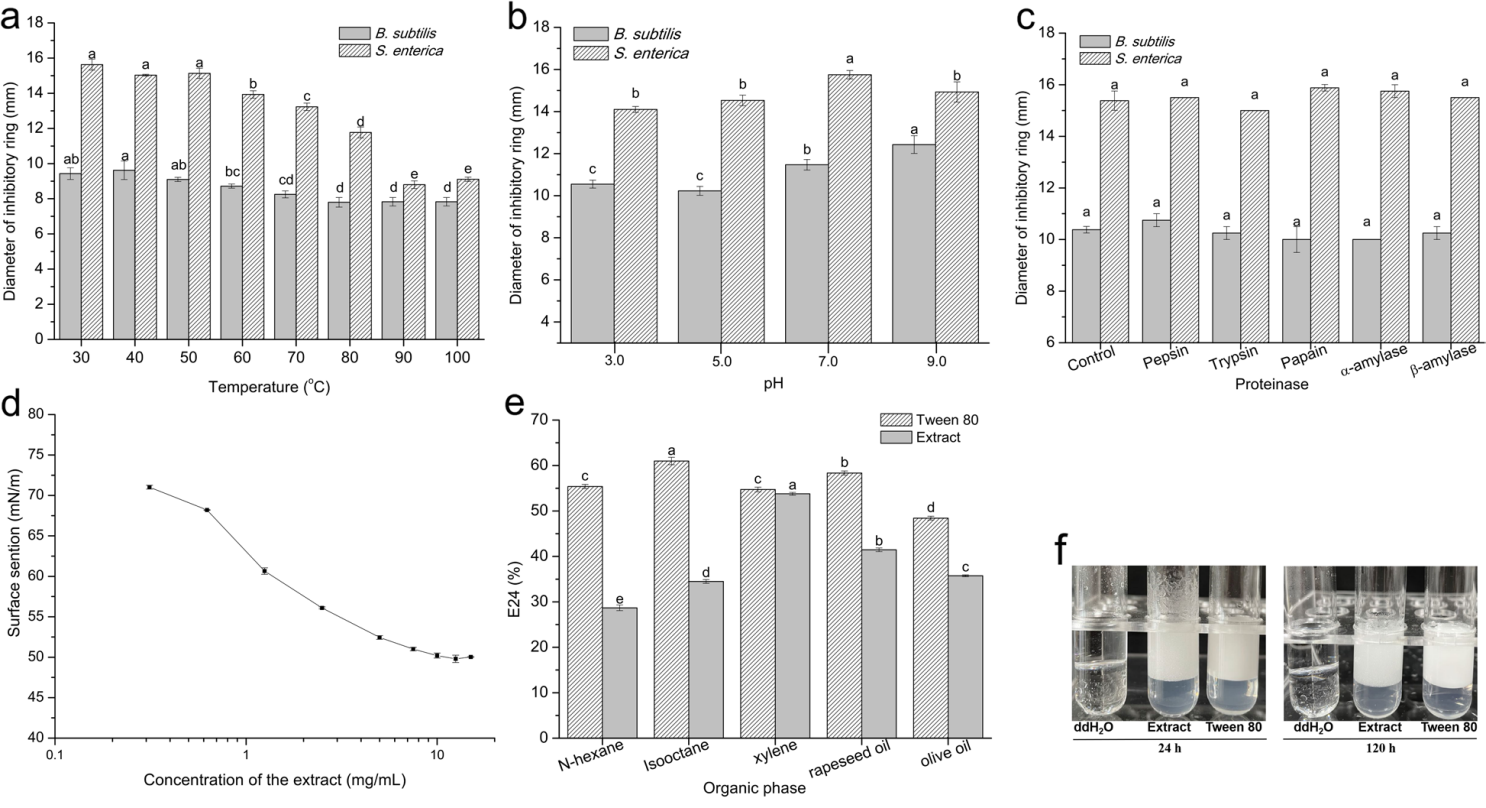
Stability and emulsifying property of the cell-surface extract. Using 15 mg/mL of the ultrasound extract, the antibacterial stability to different temperatures (**a**), pH values (**b**) and protease (**c**) were tested. And then the emulsifying characteristics of surface tension (**d**), E24 against different substrates (**e**) and emulsification stability to xylene (**f**) were evaluated

The physicochemical sensitivity of the extract to heat, pH and proteases was measured. The antibacterial activity of the extract to *B. subtilis* was slightly influenced with different temperature gradients. However, the antibacterial ability against *S. enterica* was largely reduced with temperature higher than 70 °C and the corresponding activity reduced by more than 20.57% after incubation at 80 °C and over (Fig. 4 a). Furthermore, the extract with different pH values showed similar effect tendency on the antibacterial activity against *B. subtilis* and *S. enterica*, and the antibacterial zone of the extract at pH 7.0 was slightly larger than that of the other pH values (Fig. 4 b). Unlike heat and pH, proteases played insignificant role on the antibacterial activity of the extract against *B. subtilis* and *S. enterica* (Fig. 4 c).

Moreover, the extract was evaluated for reduction of surface tension and critical micelle concentration (CMC). The result showed that the extract could reduce the surface tension from 72.708 mN/m to 51.011 mN/m associated with the concentration increased from 0.33 mg/mL to 7.5 mg/mL and then the surface tension basically kept stable even the concentration continuous increasing (Fig. 4 d). According to the logarithmic plot of the extract concentration, the CMC of 6.88 mg/mL was obtained. Besides, the extract was able to emulsify different hydrocarbon substrates, such as n-hexane, isooctane, xylene, rapeseed oil and olive oil (Fig. 4 e). The highest emulsification index (E24) of 53.78% was achieved for the xylene/extract emulsion which was equal to the emulsifying capacity of Tween80. The lowest E24 was obtained for the n-hexane oil/extract emulsion (28.69%). In addition, the E24 for xylene/extract emulsion was merely decreased by 5.77% after standing for 120 h (Fig. 4 f).

## Discussion

Foodborne pathogens and spoilage bacteria are the major cause of foodborne illnesses and cause a huge challenge to food security around the world. Most of the foodborne pathogens or spoilage bacteria are survival in the shape of biofilm by adsorbing on the biological and abiotic surfaces. Compared with planktonic cells, bacteria in biofilm are much more resistant toward antimicrobial agents, harsh environment and host immunity. Foodborne pathogens and spoilage bacteria in the biofilm shape caused critical concern for many food industries. It is attractive to develop agents with specific antibacterial and antibiofilm activity [3]. *Lactobacillus* has shown to be effective for inhibiting foodborne pathogenic bacteria with antibacterial metabolites including organic acids, bacteriocin, hydrogen peroxide (H_2_O_2_), etc. [20]. Till now, there were scarce work about cell-surface compounds of *Lactobacillus* origin with clear molecular structure and antibacterial-antibiofilm mechanism. In our lab, *L. rhamnosus* YT was isolated because its cells suspended into ddH_2_O showed distinctive inhibition zone to various Gram positive and negative foodborne spoilage bacteria on the agar plate. In this work, using the typical foodborne spoilage bacteria *B. subtilis* and *S. enterica*, the antibacterial and antibiofilm property of *L. rhamnosus* YT cells and its cell-surface substances was investigated.

The kinetic profile of the *L. rhamnosus* YT cells’s antibacterial capacity of was represented by plotting the curves of growth and the corresponding inhibitory diameter to *B. subtilis* and *S. enterica*. The antibacterial activity of *L. rhamnosus* YT cells was growth-dependent. The antibacterial substance might be composed of more than one component due to the different cultural time for the largest antibacterial diameter to *B. subtilis* and *S. enterica*. Moreover, the antibiofilm activity of the *L. rhamnosus* YT cells was detected in the LB broth because *L. rhamnosus* YT could not grow and form biofilm in this medium. The biofilm formation of the indicator strains was obviously inhibited by co-incubation with *L. rhamnosus* YT cells. These data indicated that *L. rhamnosus* YT cells had the antibacterial and antibiofilm capacity to *B. subtilis* and *S. enterica*. Moreover, the antibacterial capacity of the cell pellets increased with a constant level during the exponential growth phase and reached the peak value about 20 h after the startup of the fermentation process. The antibacterial substance was produced in a short time which would be beneficial for its production from aspects of saving energy, convenient separation, synthesis of more products in a monthly work schedule, etc.

As the antibacterial activity was closely related to the *L. rhamnosus* YT growth, the factors usually affecting strain growth were selected to further explore the relationship between growth and antibacterial activity. *L. rhamnosus* YT grow well with the biomass higher than 9.0 log CFU/mL in the broth containing various carbon and nitrogen sources. Generally, more biomass displayed higher antibacterial activity. *L. rhamnosus* YT cultivated with maltose displayed the highest biomass and the largest inhibitory zone to both *B. subtilis* and *S. enterica*. However, the difference of the biomass and parallel antibacterial activity of *L. rhamnosus* YT cultivated with various carbon and nitrogen sources was disproportional. For instance, under the circumstance of small difference in biomass, the antibacterial activity of *L. rhamnosus* YT cultivated with maltose (10.78 log CFU/mL) were obviously higher than that of with glucose (10.22 log CFU/mL) and with soybeans (10.25 log CFU/mL). Moreover, even though *L. rhamnosus* YT cultivated with glucose and soybeans displayed similar biomass and antibacterial activity to *B. subtilis,* the corresponding inhibitory zones to *S. enterica* were different. These results suggested that the carbon and nitrogen sources might affect the composition of the antibacterial substances which aimed specifically at *B. subtilis* and *S. enterica*. In many reported studies, the structure and the content of some cell-binding active compounds were significantly influenced by carbon and nitrogen sources. The total cell surface antigenicity of *L. rhamnosus* GG was increased by switching the carbohydrate source from glucose to fructose [21]. In the study carried out by Mouafo et al. [22], the biosurfactants yields of three indigenous bacterial strains (*L. delbrueckii* N2, *L. cellobiosus* TM1 and *L. plantarum* G88) with molasses or glycerol were significantly high compared to those obtained with MRS broth as substrate and the crude biosurfactants were mainly glycoproteins and glycolipids with substrate of molasses and glycerol, respectively. In contrast to carbon sources, nitrogen sources mainly affect the expression of proteins or peptides. In *L. acidophilus* NCC2628, both peptone and yeast extract had a considerable influence on the bacterial cell wall which was witnessed by changes in surface charge, hydrophobicity, and the nitrogen-to-carbon ratio. In particular, expression of the surface-layer protein was dependent on the protein source of the fermentation medium [23].

Besides carbon and nitrogen sources, the cell amounts of *L. rhamnosus* YT were constantly raised along with the increase of the initial medium pH. However, the antibacterial activity of *L. rhamnosus* YT displayed a trend of first increasing and then decreasing with pH 7.0 as a dividing point. According to these data, the yield of antibacterial substance was not continually increased accompanying with the cell growth and the most feasible initial pH value for the antibacterial activity against *S. enterica* was different from that of *B. subtilis*. Moreover, *L. rhamnosus* YT grew well at broad temperature ranges varied from 27 °C to 37 °C. The highest antibacterial activity to *S. enterica* was found at 27 °C which was lower than the optimum temperature for growth (32 °C), and temperatures higher or lower than the optimum growth temperature (32 °C) showed reduced antibacterial activity. Comparatively, the largest bacteriostatic ring to *B. subtilis* seemed to occur at condition favorable for bacterial growth. In brief, the biomass and the antibacterial capacity of *L. rhamnosus* YT could be disproportionally affected by the experimental conditions tested in this work.

The antibacterial intensity (antibacterial activity per cell) with these culture conditions was evaluated. The antibacterial intensity was disproportional to the biomass. Moreover, the antibacterial intensity against *S. enterica* was commonly higher than that of *B. subtilis*. However, when soy peptone or fish peptone was used as nitrogen source or the initial medium pH was 8.0 or 9.0, the antibacterial intensity against *S. enterica* was lower than that *B. subtilis*. These results suggested that antibacterial intensity, antibacterial composition and content could be influenced by these growth-related factors. It was in accordance with several studies that bacteriocin titers can be modified by altering the cultivation conditions of the producing bacteria and certain combinations of influencing factors [22, 24].

At present, it is paramount important for commercial exploitation to optimize factors affecting production of the antibacterial substances. Specific requirements with reference to the production of metabolites through microbial fermentation and the influencing factors may be strain dependent and could vary with different types of metabolites. The properties of the growth media including amino acid composition, carbon/nitrogen ratio, pH and lactose levels play important roles in the variation of biomass and the level of bacteriocin production [25]. In this study, the antibacterial activity of *L. rhamnosus* YT was inclined to be significantly influenced by carbon source among these factors. Particularly, *L. rhamnosus* YT cultured with rhamnose grew higher than 9.0 log CFU/mL whereas barely showed antibacterial activity. Therefore, antibacterial capacity of *L. rhamnosus* YT was tightly related to carbon source metabolism. To increase the yield of the antibacterial substance, less costly and more readily available carbon substrates should be firstly searched. From this perspective, several studies have been carried out using sugar cane molasses and glycerol as promising substrates for biosurfactants production [22]. In China, there are lots of by-products from the increasing industries of sugar cane processing, biodiesel and oleochemicals production. Therefore, it would be very interesting to carry out a test on these byproducts using *L. rhamnosus* YT strains in the antibacterial substances’ production.

Antagonistic substances isolated from the cell surface of *Lactobacillus* has been reported, such as teichoic acids from *L. plantarum* IMB19, capsular polysaccharides from *L. casei* NA-2, cell-bound exopolysaccharide from *L. fermentum* S1, chitinase from *L. rhamnosus* GG and glycolipid from *L. helvetius* M5 [26–30]. Here, combining the effects of cultural conditions and the physicochemical property of the reported cell-bound antibacterial materials, phenol and LiCl were separately used for extraction of the antibacterial substance from the cell surface of *L. rhamnosus* YT. Despite the cells lost antibacterial activity after treated by phenol or LiCl, the related extract barely performed antibacterial effects to *B. subtilis* and *S. enterica* (Fig. S1). It was speculated that the concentration of the extracted substance was not enough to perform the antibacterial function or the extraction methods were not suitable as phenol and LiCl were conventionally used to extract polysaccharide and proteins from the *Lactobacillus* surface [31, 32]. Then, ultrasonication was employed to isolate capsular material from *L. rhamnosus* YT. Firstly, the ultrasonic procedure was optimized to obtain the most of surface components and meanwhile keep the integrity of cellular structure avoiding the leakage of intracellular substances. By the optimized ultrasonic procedure, the cell pellets lost the antibacterial activity and the extracted materials displayed broad inhibitory capacity to the usual spoilage and pathogenic strains in food contamination. In addition, the antibacterial ability of the extract against Gram negative strains was much stronger than that of Gram-positive strains. It might be caused by the different cell wall composition between Gram positive and negative bacteria. Moreover, by incubation with the indicator strain, the extract exhibited a bacteriostatic mode of action against the indicator strains. Besides, the extract showed concentration dependent antibiofilm performance to *B. subtilis* and *S. enterica*. These data implied that the extract could be used as excellent candidates to control microorganism and biofilm pollution in food industry.

Till date, the antibacterial materials separated from the cell surface of *Lactobacillus* were biosurfactants, peptides, surface-proteins and teichoic acid, etc. [18, 33, 34]. These reported substances were different in physicochemical characteristics. Most of the bacteriostatic substances (extracellular polysaccharides, phosphopeptides, bacteriocins, etc.) possessed good inhibition ability under acidic to neutral conditions while reduced or even inactivated activity under alkaline conditions [35, 36]. In this work, the properties of the crude extract were tentatively explored to determine the identification of the extract. The inhibitory activity of the extract was sensitive to temperature higher than 70 °C while kept stable with a wide range of pH and multiple proteases treatments. Combining with the unsuccessful extraction with phenol and LiCl, the extract was probably not surface proteins, polysaccharide or teichoic acid which were the usual antibacterial substances separated from the cell surface of *Lactobacillus*. Recently, some cell-bound biosurfactants with antibacterial activity were isolated from *L. rhamnosus*. Biosurfactant derived from *L. rhamnosus* ATCC7469 exhibited a significant inhibitory effect on the biofilm formation of *S. mutans* due to down regulation of biofilm formation associated genes, *gtfB/C* and *ftf* [13]. Biosurfactants isolated from *L. rhamnosus* of human breast milk origin displayed potent antibiofilm ability by inhibiting surface attachment [37]. In this work, the emulsifying properties of the extract were evaluated which displayed favorable emulsifying property. Moreover, there were 684.63 μg/mL of polysaccharide and 120.79 μg/mL of protein in 1 mg/mL of the extract. Hence, the extract was probably a kind of biosurfactants.

So far, most of the discovered biosurfactants with antibacterial and antibiofilm activity were crude extract, and the corresponding properties varied widely among strains. There is inadequate information about the chemical composition and structure of biosurfactants derived from LAB, mainly due to their complexity [38]. Therefore, the specific antibacterial component would be purified and identified from the extract obtained in this work and its antibacterial and antibiofilm mechanism would be explored.

## Conclusion

In this study, the antibacterial and antibiofilm characteristics of *L. rhamnosus* YT cells were investigated. The antibacterial activity of the *L. rhamnosus* YT cells was varied along with various culture conditions and the antibacterial intensity (antibacterial activity per cell) was disproportional to the biomass. Furthermore, the cell-surface extract was isolated which displayed broad antimicrobial spectrum and antibiofilm capacity. The antibiofilm activity of the extract demonstrated to be bacteriostatic mode of action, concentration-dependent, stable to physicochemical treatments and having favorable emulsifying property. The main components of the extract were polysaccharide and protein. The properties of the extract indicated that it might be a kind of biosurfactant.

## Materials and methods

### Strains and growth conditions

*Bacillus subtilis* CICC10012 (*B. subtilis*), *Salmonella enterica* WX29 (*S. enterica*), *Staphyloccocus aureus* CICC10201 (*S. aureus*), *Bacillus cereus* ATCC11778 (*B. cereus*), *Escherichia coli* CICC10899 (*E. coli*), *Pseudomonas brenneri* CICC10271 (*P. brenneri*) were purchased from the China Center of Industrial Culture Collection (Beijing, China) and were cultivated in Luria Bertani (LB) broth with aeration at 37 °C. *L. rhamnosus* YT, preserved by the key laboratory of dairy biotechnology and safety control of Jiangsu province, was isolated from the feces of Bama longevity, Guangxi province, China. *L. rhamnosus* YT was inoculated in dMRS broth at 37 °C in static condition. The biomass was determined by viable counts [39].

### Preparation of ultrasonic extract

Two hundred microliter of MRS culture broth was inoculated with 6 mL of an overnight culture of *L. rhamnosus* YT and cultivated at 37 °C for 24 h. Cell pellets were collected by centrifugation (10,000 rpm, 4 °C, 10 min), washed twice with double distilled water (ddH_2_O), and resuspended with the same volume of ddH_2_O for ultrasonic treatment. The ultrasonic extract was obtained by high intensity ultrasonic liquid processor (Sonics & Materials, Inc., USA) with the specific sets (power 160 w, working 3 s and then pausing 2 s, 60 cycles). After that, bacteria were removed by centrifugation and the supernatant was obtained by filtering through a 0.22 μm filter. The filtered sterile supernatant was lyophilized with the given parameter (quick-frozen to − 50 °C, heating to − 5 °C within 2 h and then keeping for 14 h, heating to 5 °C within 2 h and then keeping for 2 h, heating to 15 °C within 1 h and then keeping for 24 h) by the lyophilizer (LGJ-50, Sihuan Scientific Instrument Factory, Beijing, China). The freeze-dried ultrasonic extract was resuspended in PBS buffer (10 mM, pH 7.0) and stored at − 20 °C.

### Measurement of antibacterial activity

*B. subtilis* and *S. enterica* were used as indicator bacteria. Antibacterial activity was determined by agar well diffusion method. Briefly, the colony of the indicator bacteria was inoculated into a tube containing 5 mL of LB medium and cultivated at 37 °C for 12 h. And then 100 μL of indicator bacteria suspension diluting to 1.0 × 10^6^ CFU/mL was used to spread on the plate which was prepared by pouring 30 ml LB agar medium into a plate of 90 mm diameter. After drying for 2 h, 7-mm-diameter wells were made in the plate using a sterile punch. Two hundred microliter of the tested sample solution (1.0 × 10^8^ CFU/mL of *L. rhamnosus* YT cells or 15 mg/mL of the extract) were added into the well and diffused at 4 °C for 4 h. Then the plate was transferred to the incubator at 37 °C for 8 h. Antimicrobial activity was determined by measuring the diameter size of the clear zone around the well (excluding the 7-mm hole).

### Co-incubation with indicator bacteria assay

*B. subtilis* and *S. enterica* were used as indicator bacteria. The ultrasonic extract at 15 mg/mL was used for co-incubation with the indicator bacteria. Using the same volume of PBS buffer (10 mM, pH 7.0) as control, 1 mL of the extract was added into LB broth containing 1% (v/v) indicator bacteria. The co-incubation was processed in a shaker at 37 °C for 10 h, during which the culture medium was sampled every 2 h. The viable bacterial counts of the indicator bacteria in every sample were performed as described [39].

### Inhibition of biofilm formation

The indicator bacteria suspension of *B. subtilis* and *S. enterica* was diluted to 1.0 × 10^8^ CFU/mL with fresh LB liquid medium. The *L. rhamnosus* YT suspension was adjusted independently to 1.0 × 10^7^ and 1.0 × 10^8^ CFU/mL with PBS buffer (10 mM, pH 7.0). And then, 100 μL of the indicator bacteria solution and 100 μL of *L. rhamnosus* YT suspension of different concentrations was added to each well of sterile 96-well microplate, and the wells with 100 μL of the indicator bacteria solution and 100 μL of PBS buffer (10 mM, pH 7.0) were used as control. In order to prevent boundary effects, 200 μL of distilled water was added to the peripheral wells of the 96-well microplate. After incubation at 37 °C for 24 h, the biofilm biomass was determined by the crystal violet staining method [40]. The inhibition of biofilm biomass was calculated as the followed formula.

$$\textrm{Inhibition}\ \textrm{rate}\ \left(\%\right)=\left(1-\frac{{\textrm{OD}}_{\textrm{sample}}}{{\textrm{OD}}_{\textrm{control}}}\right)\times 100\%$$

Likewise, 100 μL of ultrasonic extract solution with different concentrations (7.30 mg/mL, 36.50 mg/mL and 73.30 mg/mL) was used for the inhibition of biofilm formation followed the procedure above mentioned.

### Properties of ultrasonic extract

Determination of physicochemical characteristics: using PBS buffer as control, ultrasonic extract at 15. 0 mg/mL was processed by heat (treated independently at 30 °C, 40 °C, 50 °C, 60 °C, 70 °C, 80 °C, 90 °C, and 100 °C for 15 min), pH treatment (15. 0 mg freeze-dried ultrasonic extract dissolved in 1 mL PBS buffer solution with pH 3.0, 5.0, 7.0 and 9.0, respectively) and enzyme treatment (separately treated at 37 °C for 2 h with 2 mg/mL of pepsin, trypsin, papain, α-amylase and β-amylase). The corresponding antibacterial ability of the processed extract was determined by the agar well diffusion method.

Determination of surface tension: The surface tension of ultrasonic extract with different concentrations (0.33 ~ 10.00 mg/mL) was measured as previously described [41]. For this study, using ddH_2_O and ethanol as control, surface tension values (mN/m) was detected at 25 °C by a tensiometer (DCAT11, Dataphysics, Germany).

Determination of emulsifying activity: According to the previously reported method [42], using 1:1 ratio (v/v), n-hexane, isooctane, xylene, olive oil, and sunflower seed oil was added separately into the 1 mg/mL solution of ultrasonic extract. Vortexing for 2 min to obtain maximum emulsification and then setting at 20 °C for 24 h, the height of the emulsion layer (H24) and total liquid height (H) were measured. The emulsification index (E24) was calculated as (H24/H) × 100%. Water and 1 mg/mL of Tween 80 were used as negative and positive controls, respectively.

### Statistical analysis

Statistical analysis was done by SPSS 19.0 software (SPSS Inc., Chicago). Each trial was performed in triplicate and data of 3 independent experiments was statistically analyzed by one-way analysis of variance (ANOVA) and expressed as mean ± standard deviation (SD).

## Supplementary Information

**Additional file 1: Figure S1.** Antibacterial activity of *L. rhamnosus* YT cells and the cell surface extract isolated with phenol (a) and LiCl (b). *L. rhamnosus* YT cells were treated by phenol or LiCl to obtain the cell bound substance. Then the antibacterial activity of the treated cells and the substance against *B. subtilis* and *S. enterica* was evaluated respectively.

## Data Availability

All data generated or analyzed during this study are included in this published article.
