# Supplementary figures and images for: Antibacterial and antibiofilm potential of Lacticaseibacillus rhamnosus YT and its cell-surface extract
Source: BMC Microbiol. 2023 Jan 12;23:12. doi: 10.1186/s12866-022-02751-3 (PMC9835366; doi:10.1186/s12866-022-02751-3)

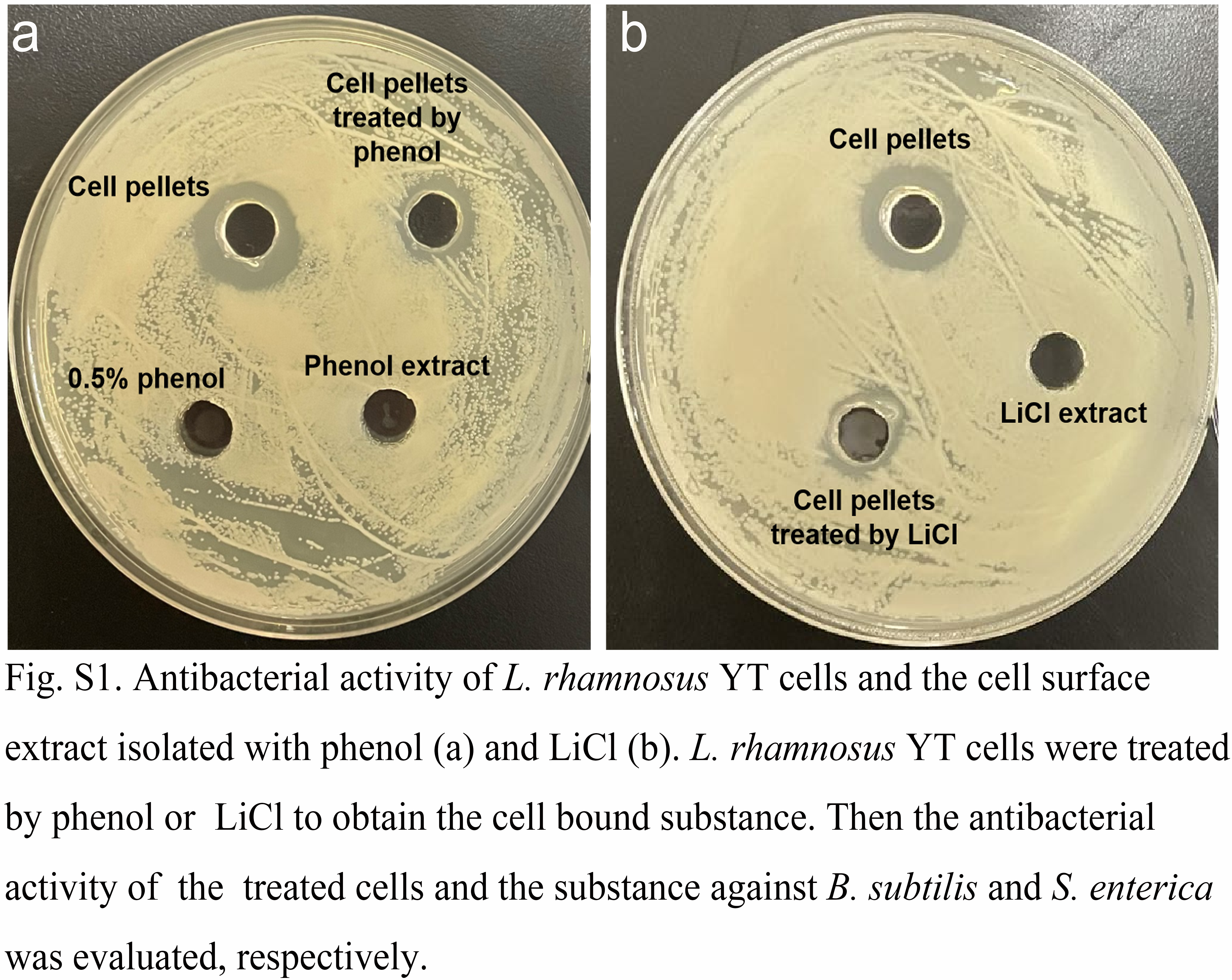

Supplement: Supplementary file 1 — Additional file 1: Figure S1. Antibacterial activity of L. rhamnosus YT cells and the cell surface extract isolated with phenol (a) and LiCl (b). L. rhamnosus YT cells were treated by phenol or LiCl to obtain the cell bound substance. Then the antibacterial activity of the treated cells and the substance against B. subtilis and S. enterica was evaluated respectively. [file 12866_2022_2751_MOESM1_ESM.tif]
